# Antiatherogenic Effect of *Camellia japonica* Fruit Extract in High Fat Diet-Fed Rats

**DOI:** 10.1155/2016/9679867

**Published:** 2016-05-31

**Authors:** Hyun-Ho Lee, Keshav Raj Paudel, Jieun Jeong, An-Jin Wi, Whoa-Shig Park, Dong-Wook Kim, Min-Ho Oak

**Affiliations:** ^1^UMR CNRS 7213, Laboratoire de Biophotonique et Pharmacologie, Faculté de Pharmacie, Université de Strasbourg, 67400 Illkirch, France; ^2^Department of Oriental Medicine Resources, Mokpo National University, Muan-gun, Jeonnam 58554, Republic of Korea; ^3^Jeonnam Forest Resources Research Institute, Naju, Jeonnam 58213, Republic of Korea; ^4^College of Pharmacy and Natural Medicine Research Institute, Mokpo National University, Muan-gun, Jeonnam 58554, Republic of Korea

## Abstract

Hypercholesterolemia is a well-known etiological factor for cardiovascular disease and a common symptom of most types of metabolic disorders.* Camellia japonica* is a traditional garden plant, and its flower and seed have been used as a base oil of traditional cosmetics in East Asia. The present study was carried out to evaluate the effect of* C. japonica* fruit extracts (CJF) in a high fat diet- (HFD-) induced hypercholesterolemic rat model. CJF was administered orally at three different doses: 100, 400, and 800 mg·kg^−1^·day^−1^ (CJF 100, 400, and 800, resp.). Our results showed that CJF possessed strong cholesterol-lowering potency as indicated by the decrease in serum total cholesterol (TC), triglyceride (TG), and low-density lipoprotein (LDL), accompanied by an increase in serum high-density lipoprotein (HDL). Furthermore, CJF reduced serum lipid peroxidation by suppressing the formation of thiobarbituric acid reactive substance. In addition, oil red O (ORO) staining of rat arteries showed decreased lipid-positive staining in the CJF-treated groups compared to the control HFD group. Taken together, these results suggest that CJF could be a potent herbal therapeutic option and source of a functional food for the prevention and treatment of atherosclerosis and other diseases associated with hypercholesterolemia.

## 1. Introduction

A high blood level of cholesterol, known as hypercholesterolemia, accelerates the oxidation of serum lipids and is known to contribute to the disruption of the circulatory systems homeostasis by a variety of chemical and physical processes. Hypercholesterolemia is generated by multiple factors such as unhealthy dietary practices, obesity, genetic disposition, and insufficient daily exercise. Previous studies have already highlighted the prevalence of hypercholesterolemia that could ultimately affect the majority of the adult population of developed countries [1]. Particularly, high blood level of low-density lipoprotein (LDL, >100 mg/dL) cholesterol is considered crucial to the pathogenesis of cardiovascular disorders (CVDs) that can lead to various disease states including atherosclerosis progression and ischemic stroke [2]. In addition, a reduced blood level of high-density lipoprotein (HDL, <40 mg/dL) cholesterol is also a critical risk factor of hypercholesterolemia-related CVDs [3]. Several studies have demonstrated that high fat or high-calorie diets can induce obesity and hyperlipidemia in the normal rodent model [4, 5].

Furthermore, various clinical studies have shown strong correlations between elevated circulating triglyceride (TG), total cholesterol (TC), and reduced HDL levels as major predictors of obesity, diabetes, and hyperlipidemia [6, 7]. The oxidation of serum lipids by oxidative/nitrative stress in the circulatory system accelerates the esterification of cholesterol and endothelium dysfunction [8]. Research evidence has revealed the increased generation of superoxide by nicotinamide adenine dinucleotide phosphate (NADPH) oxidase (NOX) activity in the hearts of cholesterol-fed Wistar rats [9]. Moreover, the cardiac expressions of antioxidant enzymes are decreased in hypercholesterolemia rats [10]. In this instance, lipid peroxidation plays a key role in vascular inflammation and leads to endothelial injury, development of atherosclerosis, and hypertension [11, 12]. Hence, the reduction of serum LDL while increasing HDL levels in blood might be a critical therapeutic target to control hyperlipidemia and obesity.

*Camellia japonica* is a popular ornamental plant that usually appears as a colony in wild environmental conditions. Geographically, it is mainly distributed in East Asia and China [13]. A previous study revealed that extracts of different parts of* C. japonica* have various biological activities such as antihuman immunodeficiency virus (HIV) activity [14], antioxidant [15], antiphotoaging [16], and hypotriglyceridemic effects [17]. However, studies on these biological activities have been focused on the seeds, flowers, and leaves, not the fruits. Our previous* in vitro* and* ex vivo* data suggested that the ethanol extract of* C. japonica* fruit (CJF) showed beneficial effects on the cardiovascular system [18]. These effects include contributing to the improvement of vascular tone by releasing nitric oxide (NO) in endothelial cells and inhibiting smooth muscle cell proliferation and migration [18].

However, the biological effects of CJF on the cardiovascular physiology are still unclear. Hyperlipidemia and obesity are known as the main causes of atherogenesis [19, 20]. Therefore, the aim of this study is to elucidate the effects of CJF on the serum lipid composition using a HFD-induced hypercholesterolemic Wistar rat model. This animal model could provide the optimum physiological conditions for evaluating the effect of CJF in hyperlipidemia and obesity. We demonstrated the effect of CJF on the serum lipid profile including the LDL, HDL, TG, and TC, as well as its inhibition of serum lipid peroxidation including lipid accumulation in the inner layer of the arteries. The results of this study along with our previous results provide evidence to support the feasibility of developing CJF as a potential therapeutic option for the treatment of atherosclerosis as well as hypercholesterolemia-associated CVDs.

## 2. Materials and Methods

### 2.1. Plant Extracts

The different* C. japonica* plant parts used in this study were collected from the southern parts of the Korean Peninsula, and a voucher specimen was deposited at the Herbarium of the Jeonnam Forest Resources Research Institute, Korea. The* C. japonica* fruit extract (CJF) was prepared as previously described [18]. Briefly, 1 kg of the dried fruit of* C. japonica* was extracted twice with 70% ethanol for 4 h, and then this crude extract was dried by using a rotatory evaporator under vacuum to obtain the final extract at a yield of 93.4 g (9.34%, w/w). For daily administration of CJF during the experimental period, various doses were freshly prepared with sterile water.

### 2.2. Animals and Oral Extract Administration

All the experimental procedures were conducted in accordance with the animal care guidelines and were approved by the relevant Committee of the Mokpo National University. Male Wistar rats (6-week-old) were purchased from Orient Bio (Seoul, Korea) and were acclimatized for 1 week with free access to water and standard rodent chow under a 12 h light-dark cycle. Then, the rats were divided into five groups (*n* = 8). The normal and control groups were fed a normal and 60% HFD diet (D12450B and D12492, Research Diet, Inc., USA), respectively, while the CJF 100, CJF 400, and CJF 800 groups were given 100, 400, and 800 mg·kg^−1^·day^−1^, respectively, in a HFD for 1 month. The formula with the diet ingredients and their energy values are shown in Tables 1 and 2. The food consumption of the rats was measured daily. The rats were housed two per cage; an equal quantity of the appropriate diet was weighed (normal and HFD) and placed in the cages of the respective groups. After 24 h, the weight of the remaining diet in each cage was measured. On the day of euthanasia, the rats were anesthetized with ethyl ether, and the blood was collected via an abdominal artery, followed by centrifugation to obtain the serum for the lipid profile analysis. The rat organs were perfused with warm phosphate-buffered saline (PBS); the arteries were isolated and then fixed with 10% formalin for the oil red O (ORO) staining assay.

### 2.3. Measurement of TC, TG, HDL, and LDL

The rat serum TC and TG levels were analyzed by using enzyme-linked immunosorbent assay (ELISA) kits (Asan Pharmaceutical Company, Seoul, Korea) according to the manufacturer's protocol. Briefly, 20 *μ*L of each of the serum samples and the cholesterol and TG standard solutions was separately mixed with 3 mL of the freshly prepared enzyme solution. The rat serum HDL level was also analyzed by using an ELISA kit according to the manufacturer's protocol (Asan Pharmaceutical Company, Seoul, Korea). Briefly, 300 *μ*L of each of the serum and separating solution was mixed and centrifuged at 3000 rpm for 10 min, and then 100 *μ*L of each of the supernatant and the HDL standard solution was mixed with 3 mL of enzyme solution. Then, the absorbance values of all the resultant colored products from the TC, TG, and HDL assays were measured within 1 h using the Epoch microplate spectrophotometer at a wavelength of 500 nm. The serum LDL level was calculated according to the Friedewald formula [21].

### 2.4. Serum Lipid Peroxidation

The serum lipid peroxidation was measured by using the thiobarbituric acid (TBA) reactive substance (TBARS) assay. First, 0.3 mL of each of the rat serum samples from each group was incubated with 50 *μ*L of 10 *μ*M copper sulfate (CuSO_4_) and 0.6 mL phosphate-buffered saline (PBS) for 4 h at 37°C. Then, 0.3 mL of this mixture was mixed with 1 and 3 mL of freshly prepared 0.67% TBA and 0.05 N hydrochloric acid (HCl), respectively. The resulting mixture was heated in boiling water for 30 min, and then the test tubes were collected and placed on ice for 5 min to stop the reaction. After cooling, the resulting chromogen was extracted with 4 mL of a mixture of 85% butanol and 15% methanol. The organic phase was separated by centrifugation at 3000 rpm for 10 min, and the absorbance was recorded at a wavelength of 540 nm using the Epoch microplate spectrophotometer. A freshly prepared malondialdehyde (MDA) solution by the hydrolysis of 1,1,3,3,-tetraethoxypropane was used as the standard. The concentration of serum TBARS was calculated by comparing with the standard curve obtained by serial dilutions of MDA [22]

(1) $$\begin{array}{c} TBARS\left(\;\mu M\;\right)=\frac{\left[A_{control}-A_{sample}\right]}{A_{control}}\times 100, \end{array}$$

where *A*_control_ and *A*_sample_ are the absorbance values of the control (standards) and serum samples, respectively.

### 2.5. ORO Staining

The ORO staining was carried out according to the protocol described by Maganto-Garcia et al. [23]. Briefly, the arteries were fixed in 5 mL of 10% neutral-buffered formalin solution overnight on a shaker at 4°C and washed overnight in PBS on a shaker at 4°C. The arteries were dehydrated at room temperature in a tube containing 5 mL propylene glycol for 2 min and then transferred to another tube containing 5 mL of 0.5% ORO for 2 to 4 h at 25°C. Then, they were washed sequentially with 85% propylene glycol in a series of three to four dishes and then further washed overnight in 5 mL PBS on a shaker at 4°C. The arteries were subsequently cut lengthwise (vertical) to expose the inner surface, fixed on silicon elastomer plates in 60 mm dishes using a syringe needle, and kept covered in PBS during the entire procedure and subsequent storage. The dish was drained, and 5 mL propylene glycol was added for 2 min to dehydrate the tissue samples at room temperature, followed by incubation with 5 mL 0.5% ORO for 2 to 4 hours at 25°C. The ORO stain was developed by washing the tissue samples with 85% propylene glycol in a series of three to four dishes. Then, the arteries were washed twice with 5 mL PBS and incubated overnight in PBS on a shaker at 4°C. A scale bar was added to show the 20 mm segments pinned next to the artery, and, finally, digital photographs of the stained arteries were taken.

### 2.6. Statistical Analysis

The statistical package for the social sciences (SPSS) software (SPSS, Chicago, IL, USA) was used to perform the data analysis. All data were presented mean ± standard error (SE). The groups were compared by using a one-way analysis of variance (ANOVA) followed by Duncan's post hoc test of multiple comparisons while *P* values ≤ 0.05 were considered statistically significant.

## 3. Results

### 3.1. Effect of CJF on Body Weight, Diet Consumption, and Liver Weight

Table 3 illustrates the effects of CJF on body weight, diet intake, and liver weight. Compared to the HFD-fed control rats, the CJF 400 and 800 groups showed a significant decrease in the final body weight. The diet consumption rate was comparable for all the groups. Although the liver weight was slightly decreased in the CJF 800 group compared to the HFD-treated group, the difference was not statistically significant.

### 3.2. Effect of CJF on Serum TC, TG, HDL, and LDL Levels

Table 4 shows the effect of CJF on the serum lipid profile of Wistar rats. Compared to the HFD group, the CJF 800 group showed a significant 2.4- and 2.3-fold decrease in TC and TG, respectively, while the HDL level increased significantly 1.8-fold. In addition, the serum LDL level was increased almost 10-fold in the HFD group compared to the normal diet group while CJF treatment decreased the serum LDL levels in a dose-dependent manner.

### 3.3. TBARS Assay and Visual Comparison of Serum Transparency

**Figure 1:**
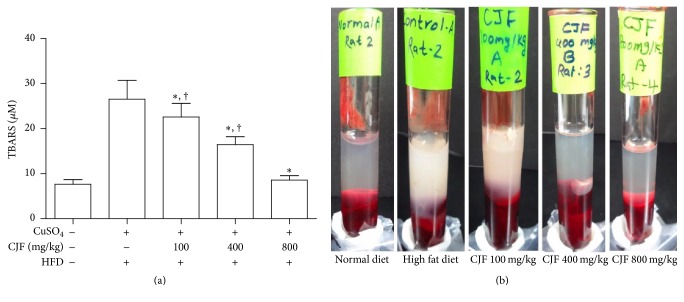
Effect of* Camellia japonica* fruits extract (CJF) on serum lipid peroxidation. (a) Measurement of thiobarbituric acid reactive substance (TBARS) level was done by comparison with the standard curve obtained using various dilutions of malondialdehyde (MDA). Values are expressed as mean ± SE (standard error), *n* = 8. ^†^ *P* < 0.05 (versus normal diet); ^*∗*^ *P* < 0.05 (versus high fat diet). HFD: high fat diet. (b) Difference in transparency of serum in each group. After centrifuge of rat blood, compared to normal diet group with transparent serum, rats fed with HFD show turbid serum due to the presence of high amount of lipid. In case of CJF-treated group, serum of rats treated with CJF 400 and CJF 800 shows clearly low turbidity with respect to HFD and their transparency is almost similar to normal diet group.

The lipid peroxidation induced by oxidizing agents is indicated by the production of MDA that ultimately binds with TBA to produce TBARS. Furthermore, MDA production is known to be a marker of the serum oxidative lipid levels, and the color of the end product of the reaction depends on the amount of lipid present in the serum. The result shows that treatment with CJF dose-dependently decreased subsequent formation of TBARS and the serum lipid accumulation (Figures 1(a) and 1(b), resp.).

### 3.4. ORO Staining of Arteries

**Figure 2:**
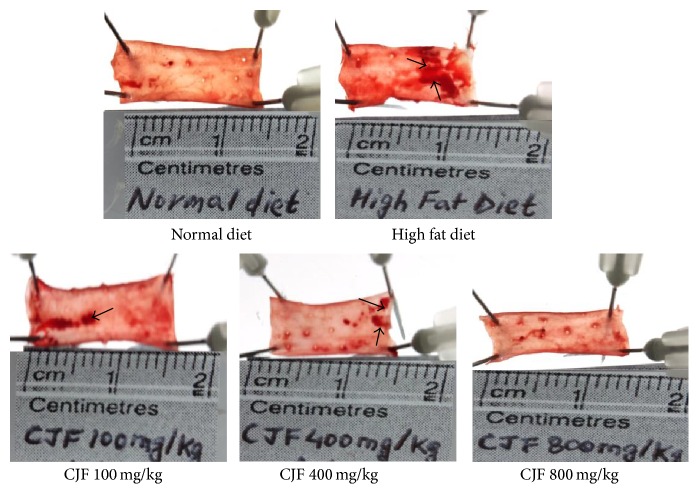
Histological analysis of oil red O staining of the rat's artery. The figure shows the photograph of ORO stained artery of Wistar rats. Scale bar beneath the artery represents the actual length of each artery. Arrow head indicates the red positive stain of ORO dye of the lipid accumulated on the vessel wall.

Figure 2 shows the effect of CJF on lipid accumulation in the arteries of Wistar rats. ORO stains the accumulated lipid in the inner layer of arteries with a dark red color and light red background. The arteries from the rats fed a normal diet showed negligible ORO staining while those on the HFD showed a significant ORO-positive staining. Therefore, treatment with CJF dose-dependently inhibited the lipid accumulation in the arteries.

## 4. Discussion

The present study demonstrated that the CJF ameliorated the discrepancies in the lipid profile of the HFD animal model by lowering the serum TC, TG, and LDL while increasing serum HDL. Moreover, the administration of CJF inhibited the lipid accumulation in the inner layer of the arteries and lipid peroxidation. Although previous studies have explored the pharmacological activity of* C. japonica* leaves and seeds, there are very few studies reporting the beneficial effects of its fruit, especially the cardiovascular actions. Therefore, our finding supports the notion that CJF could be a novel herbal source of potential drug therapies to target diseases associated with abnormal lipid metabolism.

HFD-induced hypercholesterolemic rats are used as an* in vivo* model for studying cholesterol metabolism [24, 25]. The rat model of obesity or hypercholesterolemia induced by a HFD simulates the human obesity syndrome, enabling long-term characterization [26]. Our previous study reported the vascular protective effect of the ethanolic extract of CJF* via* endothelium-dependent relaxation of porcine coronary arteries and inhibition of vascular smooth muscle cell migration [18]. In this study, we used a HFD to induce hypercholesterolemia in Wistar rats to evaluate the antiatherogenic activity of CJF. It is well established that hypercholesterolemia leads to the progression of various metabolic syndromes such as atherosclerosis, diabetes mellitus, and hypertension [27, 28]. In addition, previous studies have reported that the accumulation of blood lipids, particularly LDL, enhances the production of reactive oxygen species (ROS). Furthermore, ROS-induced oxidative stress plays an important role, at least in part, in the etiology of atherosclerosis and coronary heart disease [29, 30]. In our experimental model, the rats fed the HFD for 4 weeks did not show body weight difference compared to the normal diet. This observation suggests that the short duration of feeding with the HFD was not sufficient to induce obesity as previously reported [5, 31]. However, a HFD administered for 4 weeks successfully induced hypercholesterolemia in the Wistar rats as evidenced by the increase in serum TC and TG and decrease in serum HDL levels. Treatment with CJF for 1 month significantly reversed the abnormal cholesterol metabolism and maintained the TC and TG at levels that were comparable to those of the normal group rats (fed a standard diet). It is worth noting that CJF treatment at doses of 400 and 800 mg·kg^−1^·day^−1^ decreased the rat body weight compared to that of the control group rats fed with the HFD. This dose of CJF is within the safe dose obtained in a preliminary* in vivo* toxicity study and, therefore, indicates that the CJF could be used for its potential antiobesity effects.

The accumulated serum LDL can easily undergo oxidation by ROS. The oxidative products are then engulfed by macrophages in the tunica intima of the blood vessels and are subsequently converted into fatty streak foam cells that facilitate atherogenesis [32]. The results of the TBARS assay suggest that the CJF markedly inhibited the serum lipid peroxidation, which likely attenuated one of the crucial steps in atherogenesis. It is widely accepted that vascular homeostasis is maintained by a balanced ratio of NO and endothelin-1 (ET-1) [33]. An imbalance in NO and ET-1 results in vascular endothelial dysfunction, leading to vascular inflammation and atherosclerotic plaque formation. The function of NO depends on its level and origin. Under normal conditions, the NO produced by endothelial cells* via* endothelial NO synthase (eNOS) acts as a potent vasodilator and inhibits vascular smooth muscle cell proliferation, platelet aggregation, and leukocyte adhesion [34]. In contrast, excessive NO can generate potent oxidizing peroxynitrite anions (ONOO^−^), which react with superoxide anions to induce lipid peroxidation and endothelial dysfunction. In our previous study, CJF significantly upregulated eNOS phosphorylation in primary endothelial cells extracted from porcine coronary arteries [18]. In contrast, CJF significantly inhibited the lipid peroxidation, suggesting that it exhibited cardiovascular protective effects in this study.

During hypercholesterolemia, lipids accumulate on the inner layer of the blood vessel walls as the primary step in atherogenesis. There is a direct correlation between increased serum lipids, especially cholesterol, LDL, and other lipoprotein particles and the accumulation of lipids within the arterial wall, leading to atherosclerotic plaque development [35]. However, a high level of plasma HDL is protective against plaque formation because of its role in reverse cholesterol transport (RCT) and antiatherogenic properties [36]. A recent study indicated that high plasma HDL cholesterol levels are not solely responsible for the antiatherogenic role of HDL, but the ability of HDL to efflux the cholesterol underlies its role in RCT [37]. The result of our ORO staining revealed that administration of CJF to HFD-fed Wistar rats decreased the ORO-positive staining, which indicates the level of lipid accumulation on the artery inner layer, compared to control group (fed with HFD and untreated). Similarly, the serum HDL levels also increased. The diagnosis of hyperlipidemia by visual inspection of separated serum is often hindered by turbid serum samples due to an abnormally high accumulation of blood lipids [38]. Our results revealed that the separated serum of the HFD-fed rats showed very high turbidity compared to that of the rats fed with a normal diet. However, treatment with the CJF lowered the serum turbidity likely because of the decreased accumulation of blood lipids. There are numerous steps involved in lipid metabolism under both pathological and pathophysiological conditions. Our data suggest that CJF played a crucial role in restoring the balance of the abnormal lipid metabolism by selectively reducing the unhealthy lipids (LDL, TG, and cholesterol). In contrast, the extract increased the HDL content of the blood, which inhibited lipid peroxidation. The potential mechanism underlying the actions of the CJF could be the attenuation of the early phase of atherosclerosis and dyslipidemia, in part, due to its high polyphenol and tannin content as previously reported [39]. However, the exact underlying mechanism of CJF should be investigated further. Collectively, our results provide clear evidence that the CJF possess potential therapeutic hypercholesterolemia-lowering effects, which could prevent atherogenesis. Overall, CJF modulated cholesterol metabolism by lowering the serum TG, LDL, and TC while increasing the beneficial cholesterol (HDL).

*C. japonica* is a popular garden plant in East Asia, and its different parts including the flower, seed, and leaves are currently used as traditional medicines and commercialized as a source of cosmetics. Previous studies have indicated that the extracts of different parts of the* C. japonica* plant have various biological activities. It has been reported that extracts of* C. japonica* flowers possess antioxidant effects via scavenging of ROS and induction of antioxidant enzymes [40]. Extracts of* C. japonica* leaves have shown antihuman HIV activity by inhibiting the HIV-protease enzyme, which is required for cleaving newly synthesized polyproteins essential for virion maturity [14]. In addition, it exhibits antioxidant activity* via* free radical scavenging potency mainly due to the presence of tannins [15], antiphotoaging capability by reducing the carbonylation of tape-stripped stratum corneum after ultraviolet B (UVB) irradiation, and decreasing intracellular ROS generation in HaCaT keratinocytes [16]. Furthermore, its hypotriglyceridemic activity is mediated by decreasing serum and hepatic triglyceride level as well as lowering lipogenic enzymes activity in the liver [17]. The studies of the biological activities of* C. japonica* have been focused on the seeds, flowers, and leaves, but not the fruits. The constituents of* C. japonica* such as saponins in the seeds [41], flavonol glycosides in the leaves [42, 43], and triterpenes, several hydrolyzable tannins, acylated anthocyanins, and purine alkaloids in the flowers [44] have been reported. The present findings suggest that the fruit of* C. japonica* has strong cardiovascular protective effects and could be a good potential candidate for development as a natural-based medicine for the prevention and treatment of cardiovascular diseases. Despite these beneficial effects of the fruit, there is very little information available on its constituents compared with the seeds and leaves. Recently, Uddin et al. isolated oleanane-type triterpenes from the fruit peels of* C. japonica*, which inhibited protein tyrosine phosphatase 1B [39]. Therefore, the characterization of the chemical constituents and elucidation of the active compounds from CJF are currently being conducted by our research team.

## 5. Conclusion

In this study, we demonstrated the antiatherogenic activity of CJF mediated by the lowering of the serum TC and TG and increasing the serum HDL. Similarly, CJF inhibited the lipid accumulation on the inner blood vessel walls and significantly decreased lipid peroxidation, thereby providing evidence of its efficacy in reducing the progression of atherosclerosis. Taken together, these results suggest that CJF could be a potential herbal therapeutic option for lowering hypercholesterolemia and preventing atherosclerosis progression triggered by abnormal lipid metabolism. However, further studies are necessary to identify the active phytochemicals in CJF and elucidate their underlying vascular protective mechanisms.

## Figures and Tables

**Table 1:** Formula of diet.

Ingredient	Normal diet		High fat diet	
	gm	Kcal	gm	Kcal
Casein 80 mesh	200	800	200	800
L-Cystine	3	12	3	12
Corn starch	315	1260	0	0
Maltodextrin 10	35	140	125	500
Sucrose	350	1400	68.8	275.2
Cellulose, BW200	50	0	50	0
Soybean oil	25	225	25	225
Lard	20	180	245	2205
Mineral Mix S10026	10	0	10	0
Dicalcium phosphate	13	0	13	0
Calcium carbonate	5.5	0	5.5	0
Potassium citrate, 1 H_2_O	16.5	0	16.5	0
Vitamin Mix V10001	10	40	10	40
Choline bitartrate	2	0	2	0
FC&C yellow dye #5	0.05	0	—	—
FC&C blue dye #1	—	—	0.05	0

*Total*	1055.05	4057	773.85	4057

The amount of each ingredient in gram (gm) and corresponding energy value in kilocalorie (Kcal) for normal diet (D12450B) and high fat diet (D12492).

**Table 2:** Energy source in diet.

	Normal diet		High fat diet	
	gm %	Kcal %	gm %	Kcal %
Protein	19.2	20	26.2	20
Carbohydrate	67.3	70	26.3	20
Fat	4.3	10	34.9	60

*Total (Kcal/gm)*	3.85	100	5.24	100

The percentage of calorie of normal diet (D12450B) and high fat diet (D12492) derived from protein, carbohydrate, and fat in terms of gm% and Kcal%.

**Table 3:** Body weight, diet intake, and liver weight.

	Normal diet	HFD	CJF 100	CJF 400	CJF 800
Body weight (g)					
Initial	253.3 ± 7.4	254 ± 8.7	252.6 ± 7.2	250.6 ± 4.7	254 ± 7.9
Final	412 ± 22.5	415.8 ± 15.7	412.6 ± 13	393.9 ± 22.6	391 ± 23.7^*∗*,†^
Diet intake (g)	19.87 ± 7.24	20.78 ± 3.04	20.38 ± 3.38	19.49 ± 4.35^*∗*^	19.4 ± 3.81^*∗*^
Liver weight (g)	14.09 ± 1.79	14.46 ± 1.05	14.06 ± 1.69	14.01 ± 1.48	12.69 ± 2.15

CJF 100: Wistar rat treated with CJF extract at 100 mg·kg^−1^·day^−1^; CJF 400: Wistar rat treated with CJF extract at 400 mg·kg^−1^·day^−1^; CJF 800: Wistar rat treated with CJF extract at mg·kg^−1^·day^−1^. Values are expressed as mean ± SE (standard error), *n* = 8;  ^†^ *P* < 0.05 (versus normal diet) and ^*∗*^ *P* < 0.05 (versus high fat diet). HFD: high fat diet.

**Table 4:** Serum lipid profile.

	Normal diet	HFD	CJF 100	CJF 400	CJF 800
Values (mg/dL)					
Total cholesterol	64.03 ± 6.46	151.32 ± 17.91	119.42 ± 14.5^†^	98.57 ± 9.18^*∗*,†^	63.97 ± 6.21^*∗*^
Triglyceride	86.23 ± 26.21	187.69 ± 22.59	153.73 ± 45.25^†^	113.92 ± 47.2^*∗*^	82.75 ± 15.33^*∗*^
HDL	37.86 ± 3.59	26.78 ± 3.36	35.83 ± 1.80^*∗*^	38.63 ± 6.29^*∗*^	47.88 ± 7.98^*∗*,†^
LDL	8.92 ± 10.35	86.12 ± 19.51	53.03 ± 12.38^*∗*,†^	38.62 ± 13.2^†^	3.61 ± 6.92^*∗*^

CJF 100: Wistar rat treated with CJF extract at 100 mg·kg^−1^·day^−1^; CJF 400: Wistar rat treated with CJF extract at 400 mg·kg^−1^·day^−1^; CJF 800: Wistar rat treated with CJF extract at 800 mg·kg^−1^·day^−1^. Values are expressed as mean ± SE (standard error), *n* = 8;  ^†^ *P* < 0.05 (versus normal diet) and ^*∗*^ *P* < 0.05 (versus high fat diet). HFD: high fat diet.
